# Mild membrane depolarization in neurons induces immediate early gene transcription and acutely subdues responses to a successive stimulus

**DOI:** 10.1016/j.jbc.2022.102278

**Published:** 2022-07-19

**Authors:** Kira D.A. Rienecker, Robert G. Poston, Joshua S. Segales, Isabelle W. Finholm, Morgan H. Sono, Sorina J. Munteanu, Mina Ghaninejad-Esfahani, Ayna Rejepova, Susana Tejeda-Garibay, Kevin Wickman, Ezequiel Marron Fernandez de Velasco, Stanley A. Thayer, Ramendra N. Saha

**Affiliations:** 1 Departments of Molecular and Cell Biology, School of Natural Sciences, University of California, Merced, 5200 North Lake Road, Merced, California, USA; 2 Department of Pharmacology, University of Minnesota Medical School, Minneapolis, Minnesota, USA

**Keywords:** immediate early genes, intrinsic excitability, membrane depolarization, neuronal activity, transcription, ACSF, artificial cerebrospinal fluid, 4AP, 4-aminopyridine, Bic, bicuculline, [Ca^2+^]_i_, intracellular Ca^2+^ concentration, CHX, cyclohexamide, CREB, cAMP response element–binding protein, DMSO, dimethyl sulfoxide, ERK, extracellular signal–regulated kinase, FP, flavopiridol, IEG, immediate early gene, K^+^, potassium, KCl, potassium chloride, MAPK, mitogen-activated protein kinase, MEA, microelectrode array, NMDAR, *N*-methyl-d-aspartate receptor, pERK, phosphorylated extracellular signal–regulated kinase, PMA, phorbol 12-myristate 13-acetate, rIEG, rapid IEG, RRID, Research Resource Identifier, TBS-T, Tris-buffered saline with Tween-20, TTX, tetrodotoxin

## Abstract

Immediate early genes (IEGs) are transcribed in response to neuronal activity from sensory stimulation during multiple adaptive processes in the brain. The transcriptional profile of IEGs is indicative of the duration of neuronal activity, but its sensitivity to the strength of depolarization remains unknown. Also unknown is whether activity history of graded potential changes influence future neuronal activity. In this work with dissociated rat cortical neurons, we found that mild depolarization—mediated by elevated extracellular potassium (K^+^)—induces a wide array of rapid IEGs and transiently depresses transcriptional and signaling responses to a successive stimulus. This latter effect was independent of *de novo* transcription, translation, and signaling *via* calcineurin or mitogen-activated protein kinase. Furthermore, as measured by multiple electrode arrays and calcium imaging, mild depolarization acutely subdues subsequent spontaneous and bicuculline-evoked activity *via* calcium- and *N*-methyl-d-aspartate receptor–dependent mechanisms. Collectively, this work suggests that a recent history of graded potential changes acutely depress neuronal intrinsic properties and subsequent responses. Such effects may have several potential downstream implications, including reducing signal-to-noise ratio during synaptic plasticity processes.

In the brain, sensory stimulation and learning events alter neuronal activity and upregulate expression of immediate early genes (IEGs) and other genes, a phenomenon referred to as excitation–transcription coupling or experience-dependent transcription (1, 2, 3). Such activity-dependent transcription of IEGs is functionally important for adaptive processes, such as memory consolidation, cognitive flexibility, Hebbian plasticity, and neuronal homeostasis (4, 5, 6, 7, 8). Different modes and patterns of stimulation induce distinct gene expression programs (9, 10). In fact, the transcriptional profile of the neuron can be indicative of the duration of activity (11). We have shown that activity-induced IEG expression is characterized by three waves, including rapid IEGs (rIEGs) and delayed IEGs as well as *de novo* translation–dependent secondary response genes. Sustained neuronal activity induces rIEGs, delayed IEGs, and secondary response genes, whereas brief activity induces only rIEGs (11). These findings suggest that neurons can sense and respond to distinct activity patterns, such as its duration, with signature transcriptional programs that then likely facilitate long-term processes, such as learning and memory.

While exact roles of IEGs remain unclear in learning and memory, several IEGs—such as *Arc*, *Npas4*, *c-Fos*, and *Egr1—*are often used to demarcate active neurons allocated to a memory trace or engram during Hebbian learning (1, 5, 12, 13). Interestingly, only a subset of neurons respond to sensory stimuli and are incorporated in engrams. Usually, these neurons are marked by enhanced excitability and IEG transcription. Because the size of an engram is limited, neurons are forced to compete for allocation (12, 14, 15). This competition is partially modulated by the activity of the transcription factor cAMP response element–binding protein (CREB), which regulates certain subsets of IEGs (16, 17) and bidirectionally modulates neuronal excitability (12, 18). Overexpressing CREB enhances a neuron’s competitiveness for allocation to an engram (14, 18, 19, 20, 21, 22), whereas neurons with decreased CREB function are more likely to be excluded (12, 14). Previous activity experience also impacts a neuron’s competitiveness. A successfully allocated neuron remains excitable for about 6 h after a learning event, and during this period, it is likely to be coallocated to a new engram representing a second event (18, 20). After longer periods, allocated neurons become “refractory” or less excitable and thereby less likely to be coallocated to a second event (20). Together, it has been proposed that a neuron’s inclusion in engrams is in part determined by its intrinsic properties (19), which in turn depends on its recent history of activation (23).

The activity history of a neuron may include suprathreshold membrane polarization resulting in orthodromic or backpropagating action potentials, and also, subthreshold-graded potential changes. While action potentials are traditionally viewed as key mediators of neuronal operations, studies have shown that subthreshold-graded changes in membrane potential can effectively recruit neuronal second messengers (24), alter intrinsic properties (25), and modulate synaptic communication (26, 27, 28). Most activity-induced gene transcription studies have involved suprathreshold stimulation, including direct membrane depolarization using 55 mM extracellular potassium chloride (KCl), homeostatic potentiation of synaptic activity by prolonged tetrodotoxin (TTX) treatment followed by its washout, or disinhibition of inhibitory synapses using bicuculline (Bic) (11, 29). Other approaches have used optogenetics to mimic stimulation parameters. With these tools, Yu *et al.* (30) determined that one nuclear calcium transient induced by a single burst of action potentials is the minimum signal strength required to induce activity-dependent transcription in hippocampal neurons. However, it remains largely unknown if mild-graded potential changes are capable of triggering transcriptional responses in neurons.

In the current study, we use rat-dissociated cortical cells and low concentrations of extracellular KCl to first address whether neurons undertake IEG transcription in response to mild depolarization. We also address whether such depolarization “experience” leaves a cellular “memory” to affect future transcriptional and electrical responses. Hereby, we present data to suggest that neurons presented with mild stimulation respond transcriptionally with distinct IEG profiles for various doses of KCl. When these mildly depolarized neurons are activated again after some time, they manifest subdued transcriptional signaling and electrical responses.

## Results

### Variable doses of external KCl induces IEG transcription

**Figure 1:**
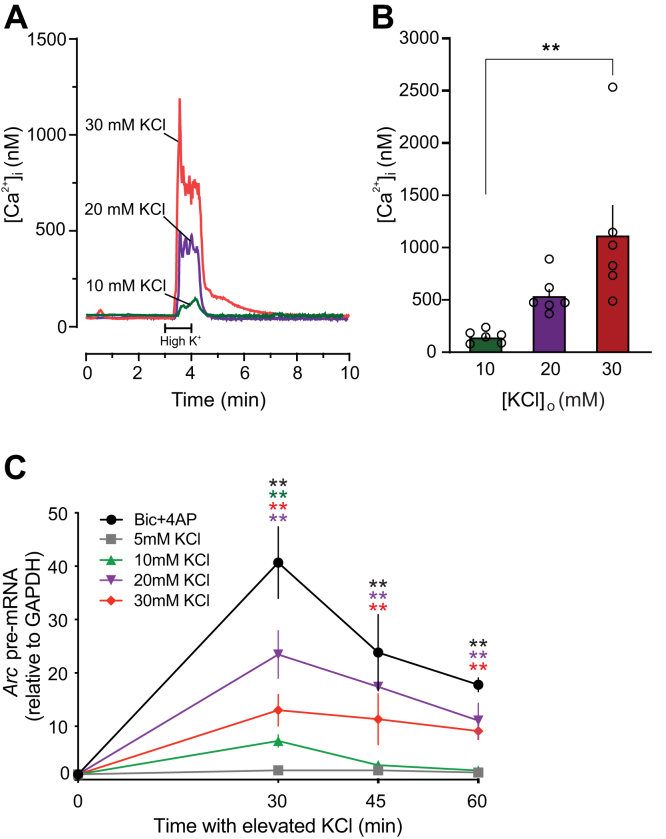
**Treatment with variable concentration of external KCl induces calcium influx and *Arc* transcription**. *A* and *B*, relation of depolarization strength to the amplitude of resulting [Ca^2+^]_i_ increase in dissociated rat neurons. *A*, representative traces from three separate recordings are overlaid to display depolarization-induced [Ca^2+^]_i_ transients. Cells were superfused with Neurobasal salt solution containing 10 mM (*green trace*), 20 mM (*purple trace*), or 30 mM (*red trace*) extracellular K^+^ at the time indicated by the horizontal bar. *B*, bar graph summarizes change in peak [Ca^2+^]_i_ evoked by the indicated concentration of extracellular K^+^ (n = 6 for all groups). Data are mean ± SEM. ∗∗*p* < 0.01, one-way ANOVA with Tukey’s post hoc test. *C*, rat neurons were treated with variable KCl for variable time, and *Arc* pre-mRNA was quantified. N = 3 to 4 for individual time points. For *Arc* pre-mRNA: interaction (*F*(16,76) = 5.842, *p* < 0.0001); TIME (*F*(4,76) = 34.97, *p* < 0.0001); and TREATMENT (*F*(4,76) = 33.19, *p* < 0.0001). One-way ANOVAs were performed for the simple main effect of TREATMENT at each level of TIME, with comparisons made to the 5 mM KCl treatment. At 30' (*F*(4,22) = 18.04, *p* < 0.0001), Bic/4AP (*p* < 0.0001), 20 mM (*p* = 0.0001), and 30 mM KCl (*p* = 0.0469) induced significantly more *Arc* pre-mRNA than 5 mM. At 45' (*F*(4,10) = 6.055, *p* = 0.0097), only Bic/4AP (*p* = 0.0077) induced significantly more than 5 mM. At 60' (*F*(4,15) = 16.30, *p* < 0.0001), Bic/4AP (*p* < 0.0001), 20 mM (*p* = 0.0038), and 30 mM (*p* = 0.0191) induced more than 5 mM. 4AP, 4-aminopyridine; Bic, bicuculline; [Ca^2+^]_i_, intracellular Ca^2+^; K^+^, potassium; KCl, potassium chloride.

We treated primary cortical neurons with variable mild KCl treatments below field standard of 55 mM KCl (31) for a minute and quantified resultant calcium influx. Cortical neurons grown on coverslips were mounted in a perfusion chamber, and intracellular Ca^2+^ concentration ([Ca^2+^]_i_) was measured using fura-2-based digital imaging as described in the Experimental procedures section. The cells were depolarized by increasing the KCl concentration in the bath, which evoked an increase in the [Ca^2+^]_i_ (Fig. 1*A*). [Ca^2+^]_i_ rose from a basal level of 45 ± 10 nM to reach a peak [Ca^2+^]_i_ that corresponded to stimulus strength (Fig. 1*B*).

Next, we investigated the downstream effect of such calcium influx on activity-induced gene transcription. Initially, we characterized the transcriptional response of our model IEG—*Arc*—over time by quantifying *Arc* pre-mRNA—not mRNA—levels as the direct readout transcriptional dynamics in response to increasing concentrations of external KCl and for comparison, also to a standard treatment protocol using Bic and 4-aminopyridine (Bic + 4AP) to induce strong synapse-based activity (Fig. 1*C*). Compared with 5 mM KCl treatment, *Arc* pre-mRNA was significantly induced by 20 mM KCl—trending similar to Bic + 4AP (32)—at 30', 45′, and 60', with a peak at 30'. About 10 mM KCl treatment induced a weak but significant response at 30′ only. Although 30 mM KCl significantly induced *Arc* at all time points, the response profile was attenuated compared with that of the 20 mM treatment (Fig. 1*C*). Noticeably, increasing the concentration of KCl did not necessarily induce more *Arc* transcription or more closely follow the Bic + 4AP induction profile.

**Figure 2:**
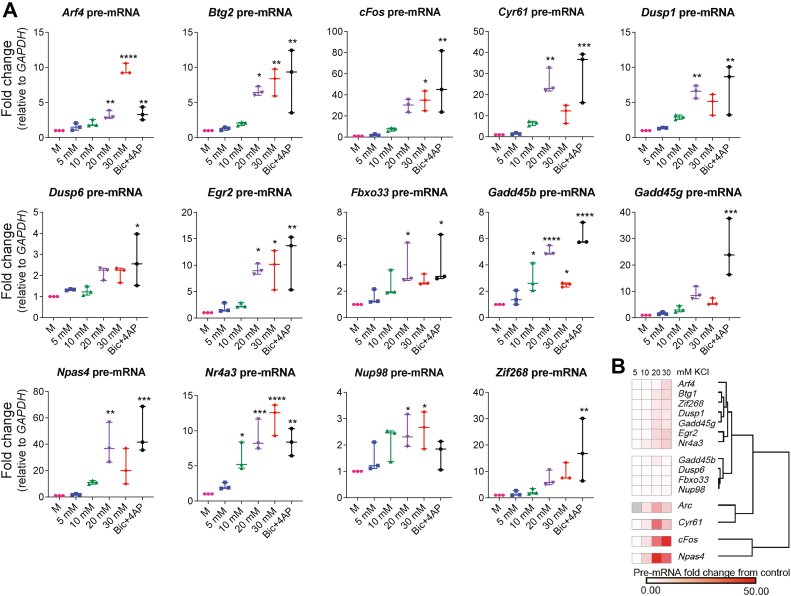
**Treatment with variable KCl induces IEGs**. *A*, samples were treated with variable KCl or Bic + 4AP (indicated on *X*-axis) for 30 min, and IEG pre-mRNA levels was quantified. N = 3. IEG induction is represented on *Y*-axis for all genes as fold change relative to the indicated housekeeping gene. All IEGs showed induction by Bic/4AP except *Nup98.* Five millimolar treatments were not significantly different to mechanical controls. ∗ indicates *p* < 0.05, ∗∗*p* < 0.01, ∗∗∗*p* < 0.001, and ∗∗∗∗*p* < 0.0001. *B*, IEGs are clustered by the similarity of their induction over all variable KCl treatments. 4AP, 4-aminopyridine; Bic, bicuculline; IEG, immediate early gene; KCl, potassium chloride.

While a few previous studies used mild KCl treatments (33, 34, 35, 36, 37, 38, 39), these publications have focused on specific IEGs, such as *Bdnf* (37) and *cFos* (39), and only a handful have used treatment times under an hour (33, 35, 37). Here, on top of *Arc* (Fig. 1*C*), we surveyed 14 additional rapidly induced IEGs—rIEGs (11)—within the hour to study their response to increasing concentration of external K^+^. After 30′ of exposure, 13 of 14 genes were significantly induced by Bic + 4AP. About 30 mM KCl significantly induced seven rIEGs, 20 mM KCl induced 10 rIEGs, and 10 mM KCl induced only two rIEGs (Fig. 2*A*). In all cases, induction under treatment with 5 mM KCl (equimolar KCl concentration as the commercial media) was not significantly different to mechanical controls. Interestingly, contrary to our initial expectation, we did not observe a linear relationship in most genes between concentration of external K^+^ and degree of transcriptional response. We then used clustering analysis to determine which IEGs responded most similarly across variable KCl treatments (Fig. 2*B*). While some IEGs were similarly induced, many showed different responses to 20 and 30 mM KCl. For instance, 20 mM KCl significantly induced *Npas4*, *Cyr61*, *Dusp1*, and *Fbxo33* above mechanical controls (M), whereas 30 mM KCl did not. We concluded that mild treatments of 20 to 30 mM KCl for 30 min are sufficient to significantly induce most IEGs, but 20 and 30 mM KCl may have overall different IEG response profiles.

### rIEG transcription and mitogen-activated protein kinase/extracellular signal–regulated kinase pathway are depressed after 1° KCl treatment in a two-step paradigm

**Figure 3:**
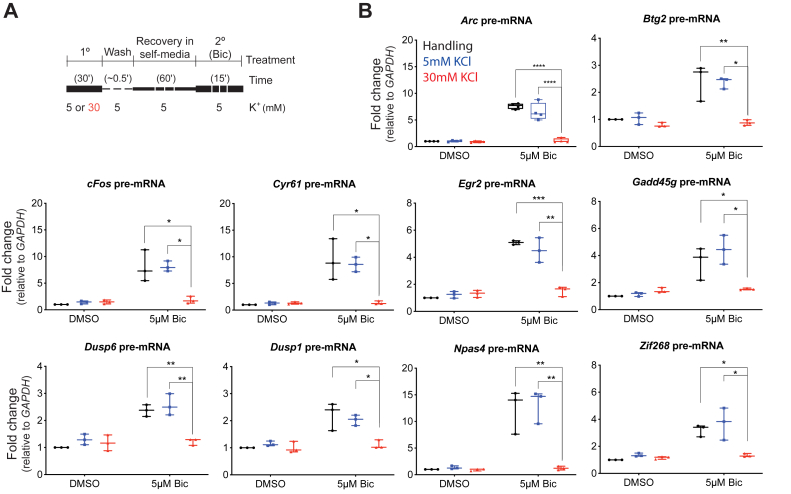
**Rapid IEGs are depressed by 1° treatment with 30 mM KCl**. *A*, the two-step treatment paradigm (1° with 5 or 30 mM KCl and then 2° with DMSO or 5 μM Bic) is visually explained. *B*, 1° treatments are color coded and explained in the *Arc* graph. 2° treatments are indicated at *X*-axis. All tested rapid IEGs showed a depression in their transcription after 1° treatment with 30 mM KCl for 30 min, compared with samples treated with 5 mM or mechanical controls. There was a significant interaction between BIC condition and KCL treatment for all genes. Significant interactions were followed up with simple main-effects analysis *via* one-way ANOVA for KCl at each level of Bic. All genes showed significant differences between 30 mM KCl treatment and M or 5 mM KCl for Bic-treated samples but not for DMSO-treated samples. N = 4 for *Arc* and N = 3 for all other genes. ∗ indicates *p* < 0.05, ∗∗*p* < 0.01, ∗∗∗∗*p* < 0.001, and ∗∗∗∗*p* < 0.0001. Bic, bicuculline; DMSO, dimethyl sulfoxide; IEG, immediate early gene; KCl, potassium chloride.

**Figure 4:**
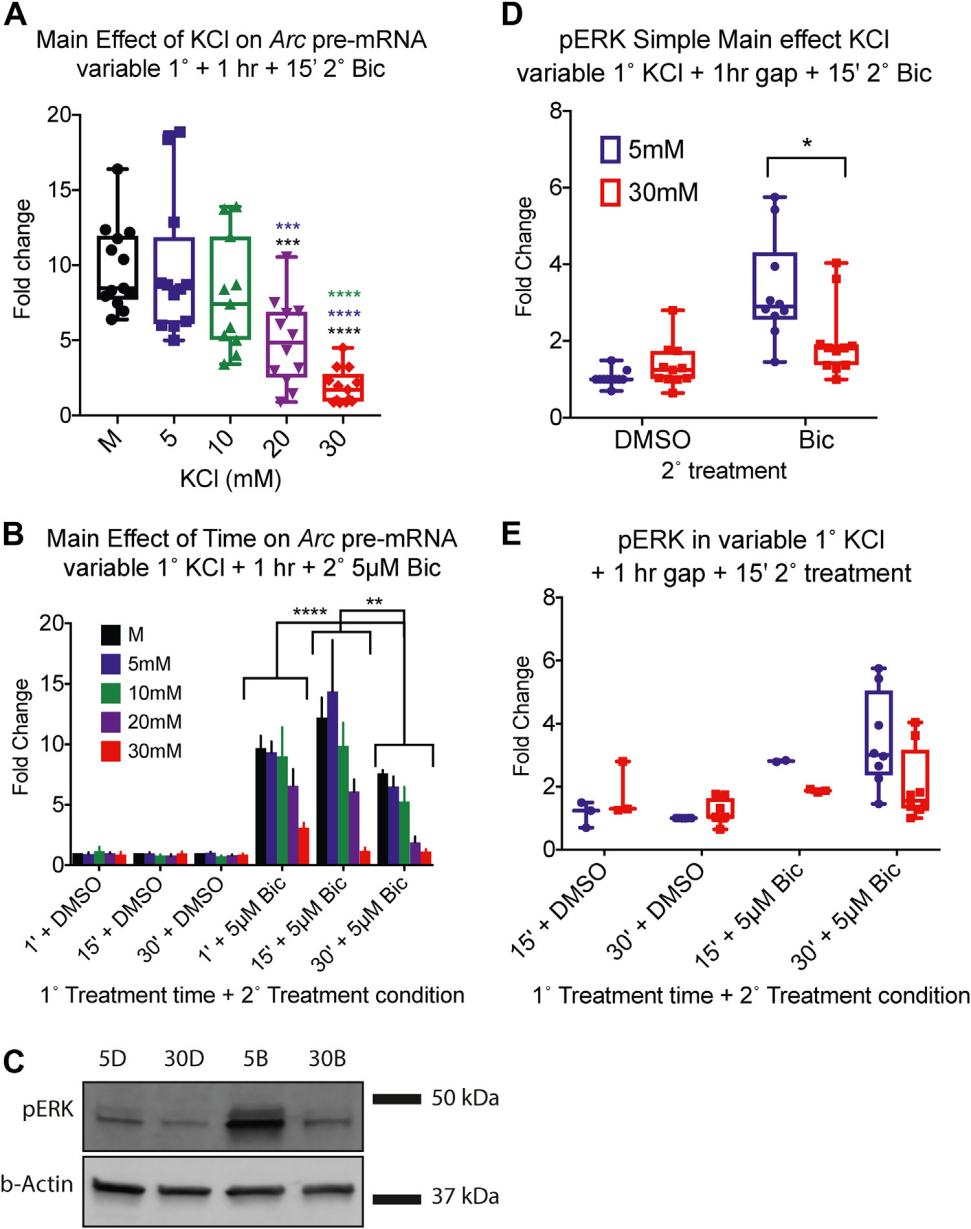
**Duration and strength of 1° treatment varies the depressive effect**. Primary neuronal culture was treated with KCl for variable 1° treatment duration before washout of 1 h and a 15′ 2° 5 μM Bic treatment. *A* and *B*, qPCR data were analyzed with two-way ANOVA. For Bic-treated samples: interaction (*F*(8,46) = 1.464, *p* = 0.1967); KCL (*F*(4,46) = 22.34, *p* < 0.0001), TIME (*F*(2, 46) = 13.50, *p* < 0.0001). N = 3 for 1′ 1° 10 mM KCl; N = 5 for 1′ 1° 20 mM KCl; N = 4 for all other KCl conditions and treatment times. *A*, the main effect of KCl for Bic-treated samples, collapsed over TIME. Samples treated with 30 mM KCl in the 1° stage induced significantly less *Arc* pre-mRNA than mechanical (*p* < 0.0001), 5 mM KCl (*p* < 0.0001), and 10 mM KCl (*p* < 0.0001). About 20 mM 1° KCl treatment also depressed *Arc* pre-mRNA compared with mechanical (*p* = 0.0002) and 5 mM KCl (*p* = 0.0005). *B*, the main effect of TIME collapsed over KCL. Bic-treated samples treated for 30′ in the 1° stage induced significantly less *Arc* pre-mRNA over all levels of KCL than samples in treated for 1' (*p* = 0.0021) or 15’ (*p* < 0.0001). There was neither significant interaction between KCL and TIME for samples treated with DMSO during the 2° stage nor were there any significant main effects. *C*, Western blot of pERK induction for 30′ 1° treatment. DMSO-treated samples induced little pERK. For 2° Bic-treated samples, 1° treatment with 30 mM KCl reduced pERK induction compared with 1° treatment with 5 mM. *D* and *E*, three-way interaction (*F*(1,35) = 0.0003, *p* = 0.9867); BIC × TIME (*F*(1,35) = 1.442, *p* = 0.2379); KCL × TIME (*F*(1,35) = 0.5238, *p* = 0.4740); BIC × KCL (*F*(1,35) = 6.323, *p* = 0.0167). N = 3 for 15′ 1°-treated samples; N = 8 for 30′ 1°-treated samples. The color key is the same for both. *D*, simple main effect of KCL collapsed over time (interaction (*F*(1,39) = 9.553, *p* = 0.0037). For Bic-treated data, there was a significant reduction in pERK levels after 30 mM KCl treatment for 30 min compared with 5 mM KCl (*p* = 0.0171). There were no significant differences in pERK level between DMSO-treated samples. *E*, total data for pERK by variable 1° treatment time. There was no significant effect of time. ∗ indicates *p* < 0.05, ∗∗*p* < 0.01, and ∗∗∗*p* < 0.001. Bic, bicuculline; DMSO, dimethyl sulfoxide; KCl, potassium chloride; pERK, phosphorylated extracellulat signal–regualated kinase; qPCR, quantitative PCR.

Next, we designed a two-step treatment paradigm to investigate the effect of mild KCl treatment on neuronal responses to subsequent stimulation (Fig. 3*A*). Primary cortical neurons were treated first with 30 mM KCl for 30' (1° KCl), which was then removed and replaced with conditioned media in a recovery step of 1 h (“wash” in schema), followed by 15 min of second round of treatment with 5 μM Bic (2° Bic) (Fig. 3*A*). We used 5 μM Bic instead of 50 μM Bic to avoid plateau effects on IEG transcription and compared 30 mM KCl pretreatment to mechanically handled (M) and 5 mM KCl controls. About 30 mM KCl was used because it induced the strongest calcium influx (Fig. 1*A*) and the strongest effect among all doses in our pilot experiments (which were later carried to term as complete experiments in Fig. 4*A*). For the read out, 10 rIEGs—including *Arc*—were selected based on their ability to be induced at least twofold by 5 μM Bic (a weaker trigger compared with Bic + 4AP). All 10 tested IEGs showed significant depression in response to the 2° Bic when 1° treatment was with 30 mM KCl (compared with 5 mM KCl or the handling control; Fig. 3*B*). These results showed that, prior treatment with 30 mM KCl depressed subsequent IEG pre-mRNA induction, even after a recovery period of 1 h.

We then asked whether the duration or strength of 1° treatment varied the depressive effect of KCl pretreatment. For these and subsequent analyses, we focused on *Arc* as a representative IEG. Primary neuronal cultures were treated with variable concentration of 1° KCl for 1', 15′, or 30' intervals, before a recovery step of 1 h and a 15′ 2° Bic treatment. Because we expect Bic but not dimethyl sulfoxide (DMSO) to induce *Arc* pre-mRNA, we broke up the data by TREATMENT and used a two-way ANOVA to analyze the effects of KCL and TIME. For Bic-treated samples, there was a significant main effect of KCL and of TIME but no interaction. Both 30 and 20 mM 1° KCl treatment showed significant depression of transcription compared with controls (Fig. 4*A*). We also found 30′ 1° treatment induced significantly less *Arc* pre-mRNA than 1′ or 15' 1° treatment (Fig. 4*B*). In contrast to Bic-treated samples, DMSO-treated samples had neither a significant interaction between KCL and TIME nor any significant main effects of KCL or TIME. We concluded that the depressive effect of 30 mM 1° KCl treatment on subsequent induction of IEG pre-mRNA persisted despite time of treatment (down to 1′ treatment), but samples with 30′ pretreatments induced overall less *Arc* pre-mRNA than 1′ or 15′ duration of 1° KCl.

Previously, we have shown that transcription of neuronal rIEGs relies on the mitogen-activated protein kinase (MAPK)/extracellular signal–regulated kinase (ERK) pathway (11). Therefore, we next investigated the impact of KCl pretreatment on the magnitude of MAPK/ERK pathway induction by assessing phosphorylated extracellular signal–regulated kinase (pERK) levels. As shown in Figure 4*C*, for Bic-treated samples, 30 mM 1° KCl significantly depressed pERK compared with 5 mM 1° KCl (quantified in Fig. 4*D*). There was no significant difference among DMSO-treated samples. Furthermore, to test any effect of 1° KCl duration on the outcome at the pERK level, we used only 15' and 30′ 1° KCl treatments (Fig. 4*E*). We performed a three-way ANOVA on the variables TREATMENT_TIME (15′ 1° or 30′ 1°), BIC (DMSO 2° or Bic 2°), and KCL (5 mM or 30 mM). There was neither significant three-way interaction nor significant two-way interactions between BIC × TIME or KCL × TIME. The two-way interaction between KCL × BIC remained significant when we consolidated the data across TIME. We concluded that 30 mM KCl pretreatment significantly depresses both *Arc* pre-mRNA and pERK induction compared with controls, across all tested 1° treatment durations.

**Figure 5:**
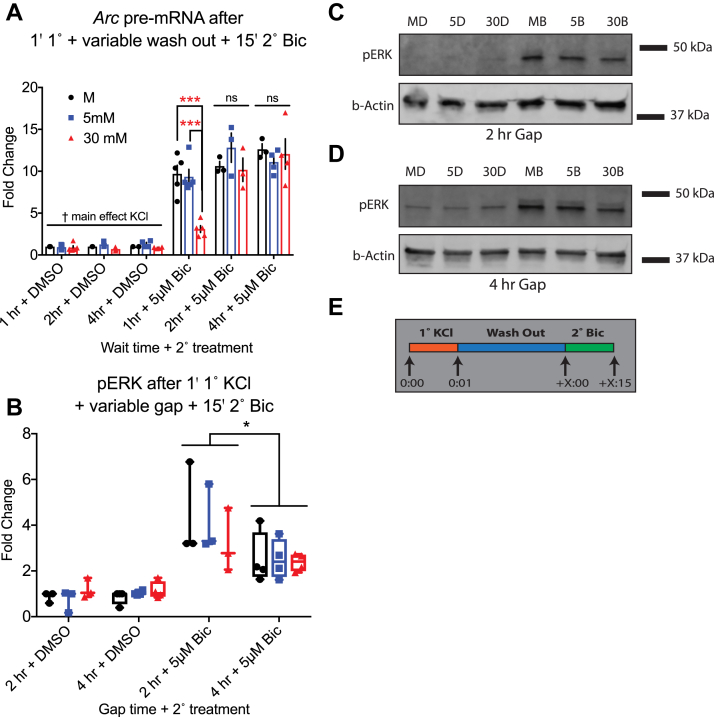
**Mild depolarization–induced depressive effects are temporary**. Resting time (“wash”) between 1 min 1° KCl treatment and 15 min 2° 5 μM Bic treatment was varied. *A*, *Arc* pre-mRNA for Bic-treated samples: interaction (*F*(4,26) = 4.470, *p* = 0.0070); simple main effect of KCL at 1 h (*F*(2,12) = 20.67, *p* = 0.0001); at 2 h (*F*(2,6) = 1.130, *p* = 0.3834); at 4 h wait (*F*(2,8) = 0.3469, *p* = 0.7170). At a 1 h wait, 30 mM 1° KCl-treated samples induced significantly less *Arc* pre-mRNA than both mechanical (*p* = 0.0003) and 5 mM KCl 1°-treated samples (*p* = 0.0004). After 2 and 4 h, this depression of *Arc* pre-mRNA had disappeared. (For DMSO-treated samples, interaction (*F*(4,25) = 1.151, *p* = 0.3561); TIME (*F*(2,25) = 0.4571, *p* = 0.6383); KCL (*F*(2,25) = 4.558, *p* = 0.0205). About 5 and 30 mM KCl-treated samples were significantly different (*p* = 0.0154). N = 5 for M, 5, and 30 mM + 1 h; N = 4 for 5 mM + 4 h and 30 mM + 4 h; N = 3 for M + 4 h, M + 2 h, 5 mM + 2 h, and 30 mM + 2 h; and N = 2 for M + 4 h DMSO sample. *B*, pERK data were analyzed: three-way interaction (*F*(2,30) = 0.4087, *p* = 0.6682); KCL × TIME (*F*(2,30) = 0.2623, *p* = 0.7711); KCL × BIC (*F*(2,30) = 1.135, *p* = 0.3349); BIC × TIME (*F*(1,30) = 6.945, *p* = 0.0132). When data were collapsed over KCL, there was a significant two-way interaction between BIC × TIME (*F*(1,38) = 7.887, *p* = 0.0078). For Bic-treated samples, overall pERK levels induced after a 2 h gap were higher than those induced after a 4 h gap (*p* = 0.0118). There were no significant differences between samples treated with various KCl concentrations either at 2 h or 4 h. N = 4 for 4 h samples; N = 3 for 2 h samples. Color key is the same as (*A*). *C*, Western blot of pERK induced after a 2 h washout step. *D*, Western blot of pERK induced after a 4 h washout step. There were no statistically significant differences between KCl conditions for either washout time. *E*, treatment diagram for variable washout time. ∗ indicates *p* < 0.05, ∗∗*p* < 0.01, ∗∗∗*p* < 0.001, and ns. Bic, bicuculline; DMSO, dimethyl sulfoxide; ns, not significant; pERK, phosphorylated extracellular signal–regulated kinase; KCl, potassium chloride.

Next, we varied the resting time between 1° and 2° treatments to discover how long the effect of 1° KCl lasted. Primary neuronal culture was treated with KCl for 1 min 1° treatment before a variable washout duration and a 2° 15′ Bic treatment. As before, we used a two-way ANOVA to analyze the effects of KCL (M, 5 mM, and 30 mM) and TIME (1, 2, and 4 h) at each level of TREATMENT (Bic/DMSO) separately. For Bic-treated samples, there was a significant two-way interaction between KCL and TIME. As before, 30 mM 1° KCl depressed *Arc* pre-mRNA compared with 5 mM and mechanical conditions for 1 min 1° treatments with a recovery period of 1 h. In contrast, there was no significant main effect of KCl for samples with 2 h or 4 h washout times (Fig. 5*A*). Only for 1 h wait time, 30 mM KCl induced significantly less *Arc* pre-mRNA than mechanical and 5 mM KCl controls, and this effect persisted even when outliers were removed.

Next, pERK data were analyzed with a three-way ANOVA to investigate the effects of KCL (5 mM or 30 mM), BIC (DMSO or Bic), and wait TIME (2 h or 4 h) for a 1 min 1° treatment. Overall, the two-way interaction between BIC × TIME was statistically significant and remained so when we collapsed the data over KCL, for which there was no significant effect. pERK levels in Bic-treated samples were statistically similar in 5 and 30 mM 1° treatment samples, both for 2 h gap and the 4 h gap (Fig. 5*B*; example blots in Fig. 5, *C* and *D*). We concluded that the depressive effect of 30 mM 1° KCl treatment on both *Arc* pre-mRNA and pERK was present after a recovery period of 1 h but disappeared by 2 h. Notably, while equivalent levels of pre-mRNA were induced after 2 or 4 h washout, overall pERK induction was significantly lower after 4 h washout. Homeostatic recruitment of signaling cascades other than MAPK/ERK (like calcineurin signaling) may explain this difference between pERK and transcription activity.

### Depressive effects of 1° KCl treatment do not recover after *de novo* transcription or translation inhibition

**Figure 6:**
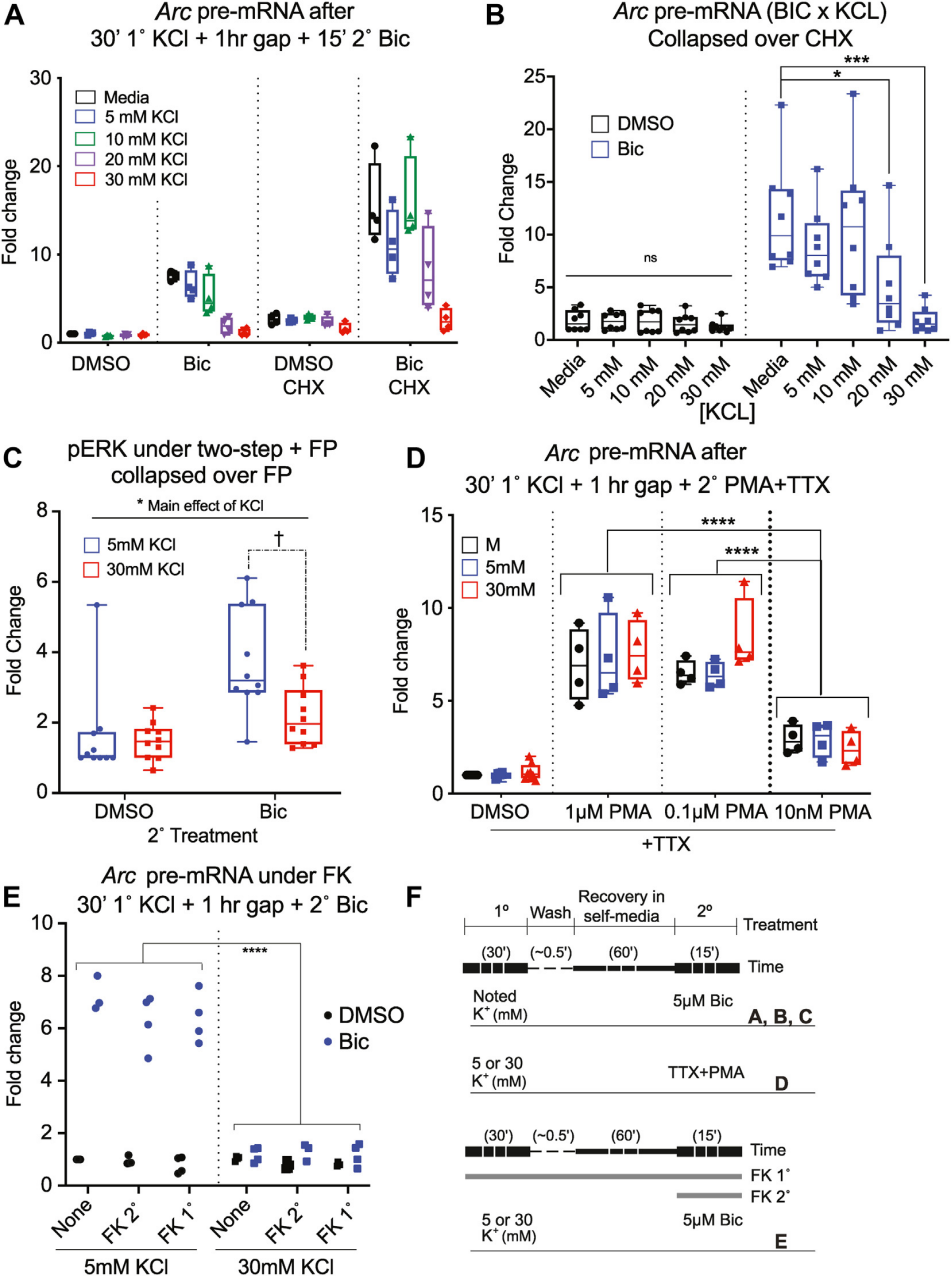
**Mild depolarization–induced depression is unaffected by translation and transcription inhibition**. *A* and *B*, samples were treated with variable 1° KCl and 2° Bic with and without the translation inhibitor CHX. N = 4 for all cells of the design. All points are displayed with minimum and maximum. Three-way interaction (*F*(4,60) = 1.870, *p* = 0.1274); CHX × BIC (*F*(1,60) = 23.14, *p* < 0.0001); KCL × BIC (*F*(4,60) = 12.25, *p* < 0.00001); KCL × CHX (*F*(4,60) = 3.260, *p* = 0.0174). There were main effects of KCL and CHX but no interaction when data were collapsed over BIC: two-way interaction KCL × CHX (*F*(4,70) = 0.7958, *p* = 0.5319); KCL (*F*(4,70) = 3.769, *p* = 0.0078); CHX (*F*(1,70) = 15.00, *p* = 0.0002). CHX elevated transcription in both DMSO and Bic conditions when data were collapsed over KCL (two-way interaction CHX × BIC (*F*(1,76) = 9.194, *p* = 0.0033); CHX(+) *versus* CHX(−) in Bic condition (*p* = 0.0003); in DMSO condition (*p* < 0.0001) (*A*). All data for the experiment are displayed. *B*, data were collapsed over CHX: BIC × KCL: interaction (*F*(4,70) = 5.192, *p* = 0.001). The simple main effect of KCl on Bic-treated samples was significant (*F*(4,35) = 6.060, *p* = 0.0008). About 20 and 30 mM KCl 1° treatment resulted in significantly less induction of *Arc* pre-mRNA at the 2° stage than the mechanical controls (20 mM [*p* = 0.0285]; 30 mM [*p* = 0.0007]). There was no significant simple main effect of KCl on DMSO-treated samples. *C*, samples were treated with and without FP during the two-step paradigm. N = 5 for all conditions. Three-way interaction (*F*(1,32) = 0.101, *p* = 0.753); FP × BIC (*F*(1,32) = 0.178, *p* = 0.676); KCl × FP (*F*(1,32) = 0.566, *p* = 0.458); KCl × BIC (*F*(1,32) = 4.250, *p* = 0.0475). When collapsed over FP, KCL × BIC was no longer significant, but main effects of KCl and BIC were (interaction [*F*(1,36) = 4.103, *p* = 0.0503]; KCL [*F*(1,36) = 5.873, *p* = 0.021]; BIC [*F*(1,36) = 16.22, *p* = 0.0003]). 5 mM KCl had significantly more detectable pERK than 30 mM KCl. Bic-treated samples had significantly more detectable pERK than DMSO-treated samples. †When outliers were removed, the two-way interaction KCL × BIC collapsed over FP remained significant (interaction [*F*(1,34) = 12.12, *p* = 0.0014]). In Bic-treated samples, there was a significant difference in pERK detected in the 5 mM KCl-treated samples compared with the 30 mM KCL-treated samples (*p* = 0.0051). *D*, samples were treated with 30' 1° KCl, 1 h washout, and 15′ 2° variable PMA to induce the MAPK pathway. N = 8 for DMSO condition, N = 4 for all PMA conditions. Two-way interaction (*F*(6,48) = 1.242, *p* = 0.302); main effect of KCL (*F*(2,48) = 1.505, *p* = 0.232); main effect of PMA (*F*(3, 48) = 125.2, *p* < 0.0001). All three PMA concentrations induced *Arc* pre-mRNA compared with DMSO: 1 μM PMA (*p* < 0.0001), 0.1 μM PMA (*p* < 0.0001), and 0.01 μM PMA (*p* = 0.0005). Both 1 μM PMA (*p* < 0.0001) and 0.1 μM PMA (*p* < 0.0001) induced significantly more *Arc* pre-mRNA than 0.01 μM PMA. Induction of *Arc* by PMA was not depressed by 1° KCl treatment. *E*, samples undergoing the two-step paradigm were treated with FK from the beginning of 1° KCL (FK 1°), the beginning of 2° Bic (FK 2°), or with DMSO (none). N = 4 for all cells except 5 mM + Bic and 30 mM FK1 + Bic, where N = 3 as values were removed because of outlying *Gapdh* values. For these values, the median value for the cell replaced the outlier to allow PRISM to perform the analysis. Three-way interaction (*F*(2,36) =1.777, *p* = 0.1837); FK × BIC (*F*(2,36) = 0.2023, *p* = 0.8178); FX × KCL (*F*(2,36) = 0.9423, *p* = 0.3991); KCL × BIC (*F*(1,36) = 366.5, *p* < 0.0001). Data were collapsed over FK treatment condition: two-way interaction (*F*(1,42) = 326.3, *p* < 0.0001). About 30 mM KCl + Bic-treated samples induced significantly less *Arc* pre-mRNA than 5 mM KCl + Bic-treated samples (*p* < 0.0001). There was no difference in induction between 30 mM KCl + DMSO and 5 mM KCl + DMSO samples (*p* = 0.574). *F*, treatment schemes for all treatments in this figure. ∗ indicates *p* < 0.05, ∗∗*p* < 0.01, ∗∗∗*p* < 0.001, and ns. Bic, bicuculline; CHX, cyclohexamide; DMSO, dimethyl sulfoxide; FP, flavopiridol; KCl, potassium chloride; MAPK, mitogen-activated protein kinase; ns, not significant; pERK, phosphorylated extracellular signal–regulated kinase; PMA, phorbol 12-myristate 13-acetate.

To understand the underlying mechanism(s) of depressive effects produced by 30 mM 1° KCl treatment, we next tested whether *de novo* translation and/or transcription are necessary for the effect. It is possible that newly translated protein is responsible for the depressive outcome during the 2° induction. New protein might be translated from *de novo* transcripts induced by the 1° treatment or from mRNA locally stored and translated at the synapse (40, 41, 42). This might result in short-term negative feedback loops, which are known to exist in IEG regulation (*e.g.*, *Sik1*) (43, 44). Therefore, we treated samples using our two-step paradigm with and without the translation inhibitor cyclohexamide (CHX). *Arc* pre-mRNA data were first analyzed with three-way ANOVA and the factors KCL (mechanical handling aka M, 5 mM, 10 mM, 20 mM, and 30 mM 1° KCl), BIC (2° Bic or DMSO), and CHX (CHX [−] or CHX [+]). Data are displayed in Figure 6*A*. The three-way interaction was not significant, but all three two-way interactions were significant. Therefore, we collapsed the data across single factors to analyze the two-way ANOVAs for simple main effects.

To determine the impact of CHX, we collapsed data first over levels of KCl. CHX (+)-treated samples induced significantly more transcription than CHX (−)-treated samples for both Bic- and DMSO-treated conditions. When we collapsed data over BIC, there was no longer a significant two-way interaction between CHX × KCL. The main effect of KCL was significant, as was the main effect of CHX. Therefore, CHX elevated overall transcription (45) but was not impacted by KCl treatment. When we collapsed data over CHX, there was a significant two-way interaction between KCL and BIC (Fig. 6*B*). As before, Bic-treated samples treated with 20 and 30 mM KCl induced significantly less *Arc* pre-mRNA than mechanical samples. There was no significant effect of KCL on DMSO-treated samples. We concluded that inhibiting translation with CHX elevated overall transcription levels but did not alter the interaction between KCL and BIC. About 30 and 20 mM 1° KCl pretreatment still depressed *Arc* pre-mRNA induction by 2° Bic stimulation and had no effect on 2° DMSO controls.

A cellular memory of mild depolarization might alternatively take the form of changes in chromatin structure or transcriptional regulation in the nucleus (2). Enhancer histone acetylation (17, 46, 47, 48) and RNA Pol II pausing (29, 49) modulate the dynamics of neuronal activity–inducible genes and could respond to previous experience. Also, RNA pol II occupancy at rIEG promoters is “poised” to initiate rapid transcription (11, 29). Impaired release or depletion of paused RNA pol II at these promoters after IEG induction by our 1° KCl treatment could depress subsequent transcription. We therefore measured pERK induction under transcription inhibition. Samples were treated with the transcription inhibitor flavopiridol (FP+ or FP−) in a similar set of two-step experiments (Fig. 6*C*). In a three-way ANOVA, there were no significant interactions involving FP. When we collapsed the data over FP and analyzed with a two-way ANOVA, there was a main effect of BIC and KCl. Across all 1° treatments, Bic-treated samples—as expected—had significantly more detectable pERK than DMSO. Across both 2° treatment conditions, 5 mM KCl had significantly more detectable pERK than 30 mM KCl. When outliers were removed from the dataset, the two-way interaction KCL × BIC was significant. In Bic-treated samples, but not DMSO-treated samples, 30 mM 1° KCl significantly depressed pERK levels compared with 5 mM 1° KCl (Fig. 6*D*). The difference between 5 mM KCl and 30 mM KCl pretreatment remained under FP treatment, suggesting that the underlying mechanisms are transcription independent. To substantiate this finding further, we required an alternative approach to determine if nuclear events (or their relevant regulatory signaling cascades) were necessary for the postdepolarization depression.

### Depressive effects of 1° KCl treatment are upstream of MEK–ERK signaling

Signaling cascades transmit information from a calcium influx across the cell membrane to the nucleus to initiate activity-induced transcription. Two signaling pathways that induce rIEGs are MAPK/ERK (50, 51) and calcineurin (52, 53, 54). Inducing these pathways independent of membrane activity or inhibiting them prior to reaching the nucleus allowed us to determine if events downstream of our manipulations (including nuclear events) were necessary for the depressive effects of 30 mM KCl pretreatments. The MAPK/ERK pathway can be induced independent of the synapse by treating cells with TTX and phorbol 12-myristate 13-acetate (PMA) (55, 56). This silences propagation of action potentials while activating PKC-dependent MAPK/ERK signaling pathway and leads to induction of IEGs (56). If nuclear events or changes in MAPK/ERK signaling downstream of PKC are the cause of IEG depression, *Arc* transcription should remain depressed when IEG transcription is activated by PMA in the 2° stage. Therefore, we applied 1 μM TTX and variable concentrations of PMA at the 2° stage instead of 5 μM Bic to discover if the depression in IEG transcription after 30 mM 1° KCl persisted.

Data obtained from the aforementioned experiments were analyzed with a two-way ANOVA for factors KCL (mechanical, 5 mM, and 30 mM) and PMA (DMSO, 1 μM PMA, 0.1 μM PMA, or 0.01 μM PMA) (Fig. 6*D*). All three PMA concentrations successfully induced *Arc* pre-mRNA compared with DMSO. There was no significant difference in induction between 1 μM PMA and 0.1 μM PMA, but 1 μM PMA and 0.1 μM PMA induced significantly more than 0.01 μM PMA. Neither there was a significant interaction between KCL and nor was there a significant main effect of KCL. Induction of *Arc* pre-mRNA by PMA was not depressed by 1° KCl treatment, indicating that the cellular “memory” of 1° KCl treatment is not “stored” downstream of PKC in the MAPK/ERK signaling pathway.

IEG transcription is also induced *via* calcineurin signaling (52, 53, 54). To determine if calcineurin signaling is necessary for the effect, we treated primary cortical cells undergoing the two-step paradigm with FK506 (FK) from the beginning of 1° KCl treatment (FK primary), the beginning of 2° Bic treatment (FK secondary), or not at all (Fig. 6*E*). FK506 blocks the activation of calcineurin (57) and significantly inhibited activity-induced IEG transcription in one of our unrelated projects (data not shown), verifying efficacy of the drug. The data in Figure 6*F* were analyzed with a three-way ANOVA for KCL (5 mM or 30 mM), BIC (DMSO or BIC), and FK (none, FK primary, or FK secondary). Neither there was a significant three-way interaction nor there were significant two-way interactions involving FK. Therefore, we collapsed the data over FK treatment to analyze KCL × BIC by two-way ANOVA. For Bic-treated samples, 30 mM 1° KCl still induced significantly less *Arc* pre-mRNA than 5 mM 1° KCl-treated samples. About 2° DMSO-treated samples showed no differences between KCl treatments. We concluded that FK506 inhibition of calcineurin signaling did not inhibit the depression of *Arc* pre-mRNA induction by 2° 5 μM Bic after 1° 30 mM KCl. Therefore, calcineurin is not necessary for the depressive effect of KCl 1° treatment.

Taken together, induction of the MAPK/ERK signaling intracellularly by PKC activation with PMA did neither replicate the depression of *Arc* induction by 30 mM KCl nor was this depression prevented by inhibition of the calcineurin signaling pathway. Signaling and nuclear events downstream of PKC and calcineurin are not sufficient or necessary (respectively) for the effect of 1° KCl treatment on a secondary stimulus an hour later. New transcription is not necessary for the depressive effect of 1° KCl on pERK in the 2° stage, as shown by the FP data.

### 1° KCl treatment acutely silences activity and depresses subsequent spontaneous and evoked bursting

**Figure 7:**
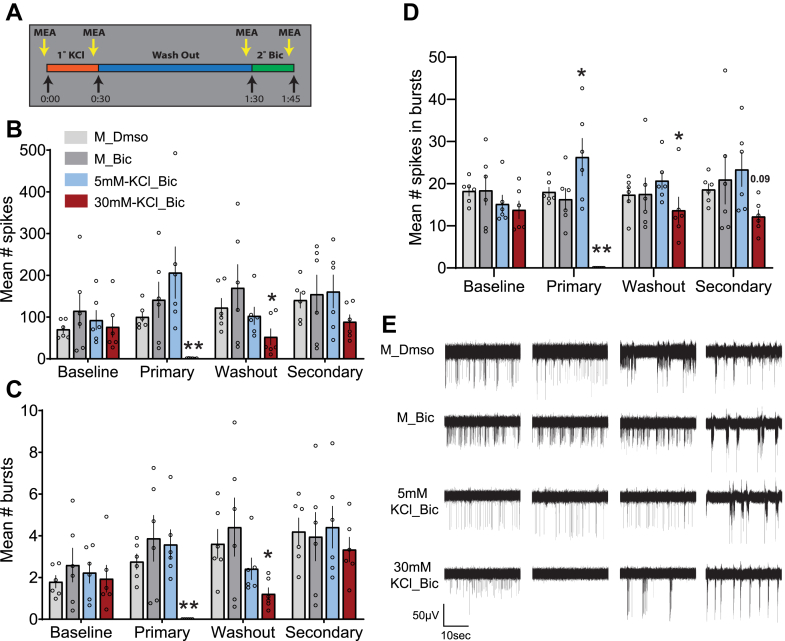
**Primary treatment with 30 mM KCl depresses neuronal firing and response to a secondary bicuculline stimulus**. The two-step KCl treatment paradigm was implemented with neurons grown on MEAs. *A*, graphical summary of KCl two-step stimulus. Two-minute recordings were conducted for each treatment group at each time point as displayed here. *B*–*D*, recordings were summarized as spike events and analyzed for several parameters. Average number of spikes (*B*), average number of bursts (*C*), and average number of spikes in bursts (*D*). *E*, example traces of recording from each treatment group and time point. Note the silencing of activity observed when cells were exposed to 30 mM KCl. All *p* values were generated with a two-way ANOVA with Dunnett’s multiple comparisons test (*B*): interaction (*F*(9,80) = 1.312, *p* = 0.2437); treatment (*F*(3,80) = 6.422, *p* = 0.0006); time point (*F*(3,80) = 1.394, *p* = 0.2507). *C*, interaction (*F*(9,80) = 1.301, *p* = 0.2494); treatment (*F*(3,80) = 5.580, *p* = 0.0016); time point (*F*(3,80) = 4.324, *p* = 0.0071). *D*, interaction (*F*(9,80) = 2.409, *p* = 0.0179); treatment (*F*(3,80) = 11.32, *p* ≤ 0.0001); and time point (*F*(3,80) = 1.101, *p* = 0.3535). Comparisons were done within each time point, comparing each treatment group to the M_Bic array average. N = 6 independent culture preparations, ∗ indicates *p* < 0.05, ∗∗*p* < 0.01, ∗∗∗*p* < 0.001. Bic, bicuculline; KCl, potassium chloride; MEA, microelectrode array.

Because the depressive effects of 1° KCl were not nuclear in nature and were upstream of PKC signaling, we next investigated whether they were related to broad measures of neuronal electrical activity. For this, we employed neuronal cultures grown on microelectrode arrays (MEAs). When the two-step stimulus paradigm was implemented on MEAs, we observed several interesting effects, summarized in Figure 7. Two-minute recordings were taken at the end of each treatment period (Fig. 7*A*). Recordings were summarized as spike time stamps, which were then analyzed for various firing-related parameters, including average spikes, average bursts, and average spikes in bursts (Fig. 7, *B*–*D*; averages taken from values for all electrodes across the recording period). Compared with the media handling control, primary treatment with 5 mM KCl enhanced the number of spikes per burst. In contrast, primary 30 mM KCl treatment completely silences detectable electrical activity for the duration of measurement. By the end of the hour-long recovery period after KCl washout, some electrical activity returns, but this remained suppressed by all measured parameters. Interestingly, the secondary Bic stimulus is still then able to elicit the expected synchronous bursting response seen in control arrays but with mildly attenuated spiking (statistically insignificant). Example traces from each treatment group and recording time point are provided (Fig. 7*E*).

**Figure 8:**
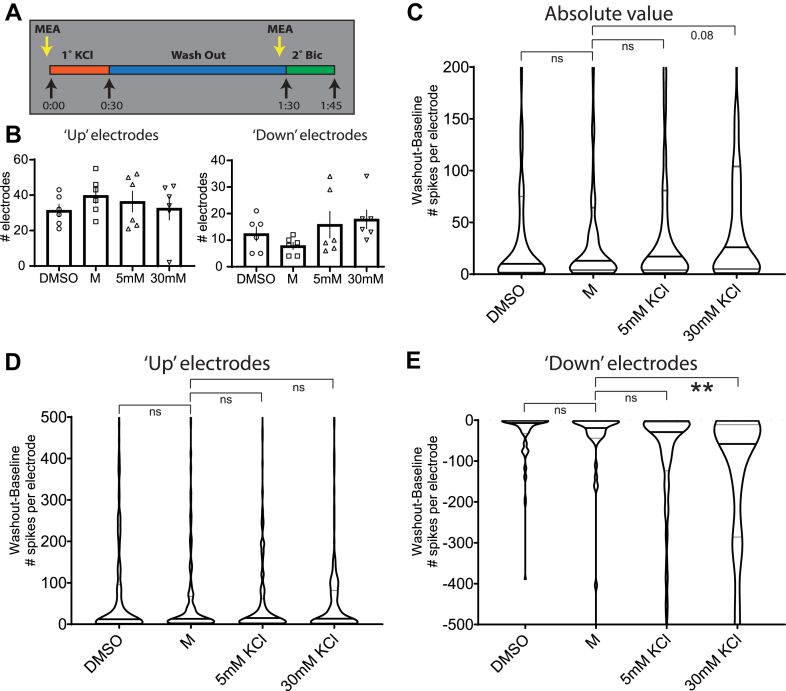
**Analysis of individual electrodes from two-step KCl MEA experiments confirms a depressive effect of primary 30 mM KCl treatment**. Because of the observed heterogeneity of results when analyzing MEA data as averages of whole recordings (Fig. 6), we considered electrodes individually (each representing a cell or group of cells) for each treatment group across all replicates. To observe the effect of the primary treatments on spontaneous firing prior to the secondary stimulus, we compared electrodes from the washout recording to baseline (washout-baseline). *A*, graphical depiction of recordings used for analysis of effects on individual electrodes. The baseline electrode values were subtracted from those recorded 1 h after washout of the primary treatment. *B*, number of electrodes classified by whether they exhibited more (up) or less (down) spiking in the washout recording compared with baseline (mean with SEM plotted). No significant changes were observed by one-way ANOVA with Dunnett’s multiple comparisons test. Electrodes up: treatment (*F*(3,20) = 0.5339, *p* = 0.6643). Electrodes down: treatment (*F*(3,20) = 1.667, *p* = 0.2060). *C*, the absolute value of individual electrode differences found between washout and baseline is plotted, with higher values indicating a greater change in the number of spontaneous spikes per electrode. *Violins* represent the distribution of all individual electrodes with detectable activity across all replicates. The *middle line* represents the median and together with the other *two lines* denotes the distribution quartiles. N = 1161 individual electrodes. Kruskal–Wallis statistic = 15.14, *p* = 0.0017. *D*, electrode differences that were positive, N = 838 individual electrodes. Kruskal–Wallis statistic = 0.4164, *p* = 0.9368. *E*, differences in electrode spiking that were negative, N = 323 individual electrodes. Kruskal–Wallis statistic = 37.50, *p* ≤ 0.0001. Statistical tests for *C*–*E* were nonparametric Kruskal–Wallis tests with Dunn’s multiple comparison. N = 6 independent culture preparations, ∗∗ indicates *p* < 0.01. KCl, potassium chloride; MEA, microelectrode array.

There are certain limitations to the initial analysis method described previously. Averaging each recording across all electrodes could mask potential diverse responses at the level of individual electrodes (representing individual cells or groups of cells). This limitation may explain the sometimes-large variation in the measured firing parameters (Fig. 7) and the lack of power to statistically confirm effects of 30 mM KCl during the secondary stimulus. To address this, we reanalyzed the same data but considered all electrodes individually. We initially employed this approach to investigate the effect of primary KCl treatment on spontaneous firing 1 h after KCl washout (Fig. 8). To do so, the number of spikes for each electrode was normalized to the baseline (washout-baseline, Fig. 8*A*). We then classified electrodes as having more or less activity after primary treatment (“up” or “down,” respectively) and determined the average number of such electrodes for each replicate (Fig. 8*B*). There were no significant differences; each treatment group consistently exhibited a similar proportion of electrodes with altered spontaneous spiking after primary KCl treatment or handling (in the case of the DMSO and M controls). Electrodes that exhibited no activity during the baseline and washout recordings were removed from the analysis (Fig. 8). The magnitude of the changes in activity is represented as absolute difference, with the distribution of all electrodes, their median, and quartiles plotted in Figure 8*C*. Here again, we found no significant changes, though 30 mM KCl treatment trended toward a greater difference in spiking from controls. However, when we considered “up” or “down” electrodes separately (Fig. 8, *D* and *E*), we observed a clear and significant attenuated effect for ”down” electrodes after 30 mM KCl treatment (Fig. 8*E*). Together, while there were similar proportions of electrode responses across treatment groups and no change in the magnitude of positive responses, “down” electrodes—with less activity—were more suppressed when treated with 30 mM KCl.

**Figure 9:**
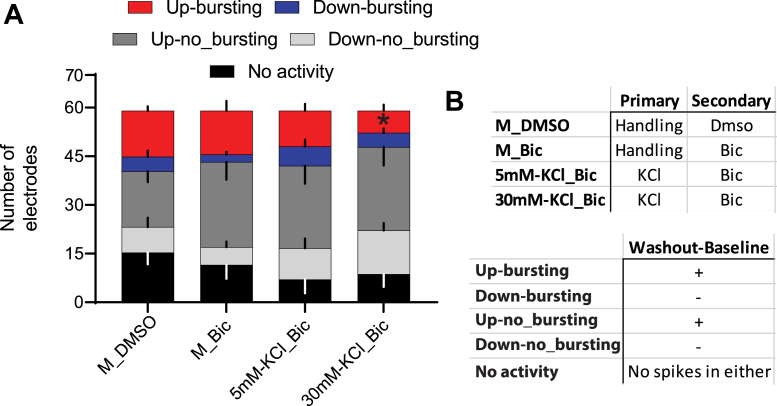
**Electrodes exhibiting elevated spiking during washout are biased toward bursting during secondary bicuculline stimulus. Primary treatment with 30 mM KCl reduces this bias**. After classifying electrodes as exhibiting increased or decreased spiking because of primary treatment (Fig. 7), we asked what happens to these electrodes during secondary bicuculline stimulus. *A*, the average distributions of electrode activity during secondary bicuculline treatment, broken down by status during the washout period and by whether the electrode subsequently displayed bursting activity at the time of secondary recording. *B*, tabular descriptions of labels for individual electrode data groupings referenced in (*A*) by treatment (*upper*) and washout/secondary filtering (*lower*). Two-way ANOVA with Dunnett’s multiple comparisons test was conducted to generate *p* values. Interaction (*F*(3,40) = 2.190, *p* = 0.1042); treatment (*F*(3,40) = 1.397, *p* = 0.2578); washout difference (*F*(1,40) = 27.08, *p* ≤ 0.0001). Each filtered category (upbursting, etc) was compared with the corresponding M_Bic group. N = 6 independent culture preparations. ∗ indicates *p* < 0.05. Bic, bicuculline; KCl, potassium chloride.

With electrodes now classified by effect of primary treatment on spontaneous spiking (“up” and “down”), we explored the behavior of these electrode groups during secondary Bic stimulus. Given that Bic treatment induces recurrent synchronous bursting, we further classified individual electrodes by whether they detected bursting after Bic treatment (for clarity, sample designations and filtering are detailed in Fig. 9*B*). Figure 9*A* displays the average number of electrodes under each classification for each treatment group. Under control conditions, electrodes that respond to Bic with bursting tend to have also had elevated activity during the washout period. Interestingly, when cells were exposed to 30 mM 1° KCl, this biasing is significantly reduced and fewer electrodes exhibit bursting regardless of their washout classification. Overall, 30 mM KCl treatment silences neuronal activity during its application (Fig. 7, *B*–*E*), suppresses subsequent spontaneous activity after washout (Figure 7, Figure 8*E*), and attenuates Bic-evoked activity in the form of bursting (Fig. 9*A*).

### 1° KCl treatment effects are calcium and *N*-methyl-d-aspartate receptor dependent

**Figure 10:**
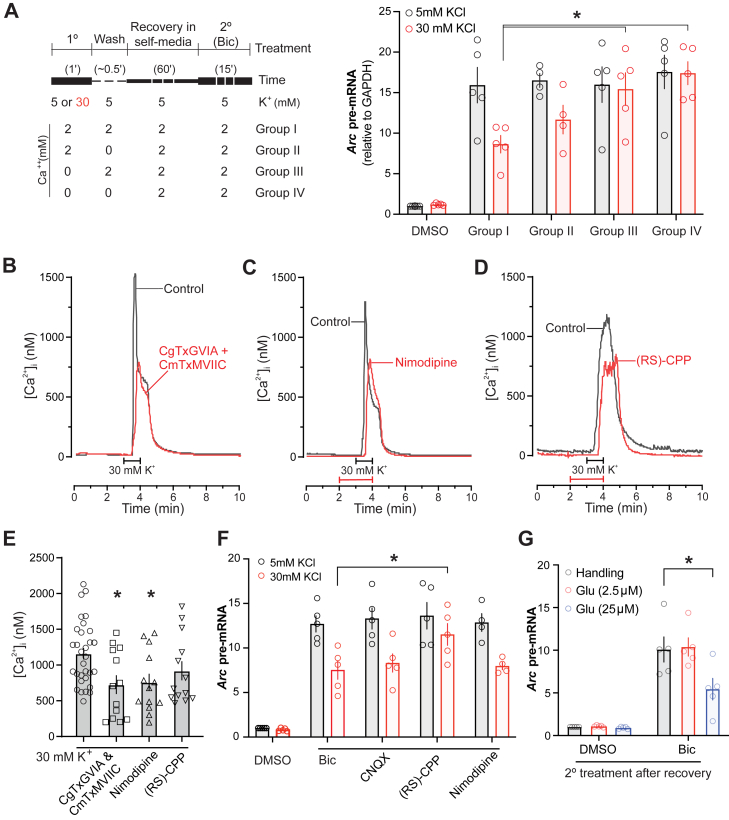
**Primary treatment with 30 mM KCl induces depression *via* calcium influx and NMDAR activation**. *A*, rat neurons were treated in the two-step experiment with or without calcium during the 1° 30 mM KCl treatment (group I–IV) and then subjected to the 2° synaptic activation (5 μM bicuculline [Bic]) an hour later. Using one-way ANOVAs and post hoc comparisons using Tukey’s correction for multiple comparisons showed a significant difference between DMSO and all other treatment conditions (using 2° Bic; *p* < 0.0001). There was also a significant difference between Bic in group I *versus* Bic in group III (Ca^2+^-free during the 1° treatment only; *p* = 0.0187) and group IV (Ca^2+^-free during the 1° and wash stages; *p* = 0.0021). *B*–*D*, representative traces show pharmacological characterization of depolarization-induced [Ca^2+^]_i_ transients. [Ca^2+^]_i_ transients from two separate recordings are overlaid to display responses evoked by 30 mM K^+^ in the absence (*black trace*) or the presence (*red trace*) of the indicated Ca^2+^ channel inhibitors. The recording chamber was superfused with 30 mM K^+^ at the time indicated by the *horizontal bar*. *B*, cells were preincubated with 1 μM CmTx MVIIC and 1 μM CgTx GVIA in combination for 1 h during loading with fura-2 AM. These toxins are essentially irreversible over the time course of this experiment (84) and thus were not present in the bath during recording. About 10 μM nimodipine (*C*) or 10 μM (RS)-CPP (*D*) was applied to the recording chamber 2 min prior to and during stimulation as indicated by the *red horizontal bar*. *E*, bar graph summarizes change in peak [Ca^2+^]_i_ evoked by 1 min superfusion with 30 mM extracellular K^+^ in the absence (control; n = 31) or the presence of the indicated treatments (n = 13 for all treatment groups). Control recordings were run in parallel with drug-treated cohorts; controls were not significantly different across cohorts and thus pooled. Data are mean ± SEM. ∗*p* < 0.05 relative to control, one-way ANOVA with Tukey’s post hoc test. *F*, neurons were treated in the two-step experiment with or without indicated washable inhibitors of voltage-gated calcium channels during the 1° 30 mM KCl treatment and then subjected to the 2° synaptic activation an hour later. Using one-way ANOVAs and post hoc comparisons using Tukey’s correction for multiple comparisons showed a significant difference between 2° Bic without inhibitors and 2° Bic with 10 μM RS-CPP (∗*p* = 0.0339). Similar comparisons of 2° Bic with 2° Bic and 10 μM CNQX or 10 μM nimodipine were not significant. *G*, neurons were treated in the two-step experiment where the 1° treatment was with indicated doses of glutamate, which was then followed by the 2° synaptic activation an hour later. ∗*p* < 0.05; similar statistical analyses as in (*F*) were performed. [Ca^2+^]_i_, intracellular intracellular Ca^2+^ concentration; CgTx GVIA, conotoxin GVIA; CmTx MVIIC, conotoxin MVIIC; DMSO, dimethyl sulfoxide; KCl, potassium chloride; NMDAR, *N*-methyl-d-aspartate receptor.

Next, to unveil an underlying mechanism, we asked if the suppressive effects of subthreshold depolarization are mediated by depolarization-dependent calcium influx. We performed the two-step-experiment, now in Ca^2+^-containing or Ca^2+^-free artificial cerebrospinal fluid (ACSF) during the 1° treatment. Cells were either treated in the continued presence of Ca^2+^ throughout (group I, Fig. 10*A*), or, in Ca^2+^-free ACSF during the KCl treatment and/or the subsequent washouts (groups II–IV, Fig. 10*A*). After an hour of recovery, the suppressive effects of 30 mM KCl were significantly neutralized in cells that were treated without Ca^2+^ in the 1° round (Fig. 10*A*). The absence of Ca^2+^ during the 1° 30 mM KCl treatment effectively recovered *Arc* transcription during the 2° Bic treatment to the level seen in neurons with 1° 5 mM KCl treatment. This finding suggests that depolarization-induced calcium influx is necessary for subsequent suppressive effects.

Next, to delineate possible routes of 1° depolarization-induced calcium influx, we imaged [Ca^2+^]_i_ in neurons treated with 30 mM KCl in the presence of pharmacological inhibitors of various Ca^2+^ channels. Consistent with a previous report (58), depolarization-evoked Ca^2+^ influx was primarily mediated by voltage-gated Ca^2+^ channels. An hour long pretreatment with 1 μM ɷ-conotoxin MVIIC and 1 μM ɷ-conotoxin GVIA in combination inhibited significantly the peak of 30 mM KCl-evoked [Ca^2+^]_i_ increase by 36% (Fig. 10, *B* and *E*), suggesting Ca^2+^ influx is mediated by N-, P-, and Q-type Ca^2+^ channels. Treatment with 10 μM nimodipine for 2 min prior to and during the application of 30 mM KCl inhibited significantly the amplitude of the response by 30%, suggesting the involvement of L-type Ca^2+^ channels (Fig. 10, *C* and *E*). Because N, P-, and Q-type voltage-gated Ca^2+^ channels mediate Ca^2+^ influx that triggers the release of the neurotransmitter glutamate (59), we also determined the role of *N*-methyl-d-aspartate receptors (NMDARs), possibly activated by depolarization-evoked glutamate release, on the 30 mM KCl-evoked increase in [Ca^2+^]_i_. Treatment with 10 μM (RS)-CPP, a competitive and reversible antagonist of the NMDAR, did not significantly attenuate the 30 mM KCl-evoked increase in [Ca^2+^]_i_ (Fig. 10, *D* and *E*) (*p* = 0.39). Note that these [Ca^2+^]_i_ recordings focused on the soma and therefore do not include or infer about any changes in highly localized [Ca^2+^]_i_ increases in dendritic spines.

To relate some of these findings to our two-step transcriptional assay, we performed the later with several pharmacological inhibitors for voltage-sensitive calcium channels (Fig. 10*E*). Because the readout of the two-step assay relies on Ca^2+^-dependent excitation–transcription coupling (*Arc* transcription), we were limited to reversible inhibition of calcium channels and could not use the irreversible conotoxin inhibitors. Contrary to our expectation, nimodipine and CNQX, the AMPAR inhibitor, failed to reverse the 30 mM KCl-induced suppression of 2° *Arc* transcription. Instead, blocking of the NMDAR with (RS)-CPP during the 1° 30 mM KCl treatment significantly attenuated the inhibitory effect (Fig. 10*F*). Taken together, these findings suggest that 30 mM KCl 1° depolarization leads to presynaptic Ca^2+^ influx *via* N-, P-, and Q-type Ca^2+^ channels, followed by synaptic discharge of glutamate and activation of postsynaptic NMDARs that then *via* downstream postsynaptic signaling may cause the depression. To test the last part of this hypothesis, we performed an experiment where the 30 mM KCl 1° treatment was replaced with 1° treatment with various concentrations of glutamate. As expected, when neurons were treated with 25 mM glutamate for 1 min (but not 2.5 mM glutamate), we recorded a depression in the 2° transcriptional response (Fig. 10*G*). Together, these datasets suggest that elevated K^+^ 1° treatment leads to depressive 2° responses *via* both presynaptic and postsynaptic components, which include voltage-gated Ca^2+^ influx and NMDARs.

## Discussion

The activity history of a neuron impacts downstream neuronal functions (11, 23). Such history could include subthreshold-graded potential changes, but effects of such mild activity remain largely unexplored. In our current study, we found that mild depolarization with low concentrations of extracellular KCl (1) induced rIEG transcription, (2) depressed subsequent transcriptional signaling (pERK), and electrical responses to synaptic stimulation transiently, and (3) such depression relied on depolarization-induced calcium influx and engagement of NMDARs, but not *de novo* transcription, translation, or CaN signaling.

Previously, we have shown that different activity patterns induce distinct IEG transcription profiles (11). However, it remained unknown whether differing strengths of graded depolarization have an analogous transcriptional effect, if any at all. About 50 to 55 mM KCl is considered to induce full-strength depolarization in primary neurons (membrane potential reverses), with gene induction comparable to synaptic stimulation. Therefore, we used low doses of 10, 20, and 30 mM KCl to investigate whether modest depolarization differentially induced rIEGs. Such low doses depolarize the membrane potential from the resting potential up to −45 mV (60, 61, 62). All three doses were able to induce transcription, but they did not always induce the same IEGs. Furthermore, in several cases, 20 mM KCl induced transcription, whereas 30 mM did not (Fig. 1). Visual examination of our clustering diagram suggested that the relationship between KCl dose and IEG induction strength was not linear. We conclude that mild depolarization can generate robust transcription of many rIEGs, and the strength of such depolarization could trigger different rIEG transcription profiles.

In addition to induction of depolarization strength-specific profiles of rIEGs, mild depolarization also impacted responses to a secondary synaptic stimulation. Such activity history had a depressive effect on subsequent transcription and pERK levels induced at a later time. To understand the underlying mechanism of such mild depolarization–induced “cellular memory,” we have ruled out the involvement of *de novo* transcription and/or translation. In our studies, the effects of interrupting transcription were less clear. We used pERK levels as readout for this assay upstream of transcription, while blocking the later with the transcription inhibitor FP, which did not rescue pERK levels otherwise depressed by 30 mM 1° KCl treatment. FP is a potent transcription inhibitor and is not competitive with ATP (63, 64, 65), and therefore likely more specific. Furthermore, FP rapidly downregulates rIEGs, like FOS and GADD45B, within minutes of treatment (29, 63, 66). Put together, the depressive effect of 1° KCl is not dependent upon *de novo* transcription, as the stringent transcription inhibitor (FP) did not rescue pERK levels.

We took an alternative approach by determining if the cellular “memory” effecting transcriptional depression existed downstream of the MAPK/ERK and CaN signaling cascades. If a “memory” existed at the level of gene regulation, we would have replicated the synaptic activity–dependent depressive effect on IEG transcription when we induced transcription extrasynaptically with PMA (to activate PKC) and TTX (to suppress synaptic activity) treatment. Instead, PMA induced IEG transcription to similar levels for all 1° KCl conditions, suggesting the depressive effect is upstream of transcriptional events. Furthermore, CaN inhibition had no effect on the depressive effect of 1° KCl treatment, indicating CaN signaling was not necessary for the effect. Therefore, any cellular “memory” of the 1° KCl treatment likely exists upstream of PKC signaling, and not in the nucleus. Also, signaling for such memory is likely independent of the signaling cascades leading to mild depolarization–induced gene transcription shown in Figures 1 and 2.

How does 1° KCl impact overall neuronal activity? Our imaging analyses reveal a sustained level of increased calcium in the soma during the duration of depolarization. Also, MEA data revealed that 30 mM external KCl entirely silenced neuronal spikes and bursts during the application. This effect was curious but could be explained by refraction of voltage-gated channels. After washout, neurons regained activity by the end of the recovery stage of the two-step paradigm, but total spikes, bursts, and spikes per burst remained depressed. During the 2° Bic stimulus, these properties recovered entirely, though spikes per burst trended to depression. Interestingly, after the washout, we noticed a dichotomous response from our electrodes. Compared with baseline after 1° KCl treatment washout, activity on some neurons increased (“up”), whereas in others, it decreased (“down”). Similar duality in responses after KCl treatment has been reported previously (60). Interestingly, in the 30 mM KCl condition during the 2° stimulus, the “up” electrodes experienced attenuated bursting in the 2° stage compared with controls. This depression in activity is not likely because of decreased cell viability by the 2° stage because of the following reasons. First, we observed transcription depressed by 1′ of 30 mM 1° KCl treatment and 1 h washout recovered after 2 h (longer 1° KCl treatments may very well have longer lasting effects); second, total spikes, bursts, and spikes per burst recovered during the 2° stage; Third, KCl treatments between 25 and 40 mM KCl are known to have a protective effect on neuronal viability (67).

Finally, aforementioned depressive effects were hypothesized at this point to be mediated by synaptic processes. Our data indicate that both presynaptic calcium influx and postsynaptic NMDAR engagement is involved in the process. Mild depolarization leads to calcium influx through presynaptically abundant N-, P-, and Q-type Ca^2+^ channels, causing synaptic glutamate release. Such glutamate likely then activates postsynaptically abundant NMDARs leading to depression. Activation of NMDARs is a key step in the process as indicated by the finding that bath application of glutamate is sufficient to induce the depression subsequently. This sufficiency also indicates that the mechanism is likely not driven by presynaptic vesicle exhaustion. Potentially, in addition to NMDARs, additional molecular mechanisms, such as metabotropic glutamate receptors, may be involved in parallel (68).

Our investigation here has led us to suspect that K^+^-treated neurons may be transiently tuning intrinsic excitability. Potential mechanisms of this transient effect include reorganization of membrane-associated structures. For instance, the axon initial segment, a specialized neuronal subcompartment localized at the beginning of the axon, is linked to modulation of intrinsic excitability (33, 36, 69). Prolonged global depolarization by KCl shifts the axon initial segment away from the cell body in an L-type channel and CaN-dependent manner and decreases neuronal excitability (33). Many signaling pathways that respond to KCl-mediated depolarization also regulate intrinsic plasticity in response to ongoing activity (36). Also, the distribution and phosphorylation status of ion channels like the L-type channels alter the firing patterns of the cell (3, 25, 70). Interestingly, CA3 neurons in organotypic cultures modulate their intrinsic firing pattern depending on their history of ongoing subthreshold activity and kinase activity (25). Here, organotypic cultures of rat CA3 neurons experienced paired pulses applied at 1 Hz and repeated 500 times for a total of ∼8 min. This subthreshold conditioning did not require the cells to fire but elicited long-lasting changes in the discharge dynamics of these neurons. This effect was reproduced using stimulation with intrasomatic injection of subthreshold depolarizing pulses and separately in acute slice preparations. Importantly, the conditioning effect was blocked by adding PKA and PKC inhibitors, suggesting the changes are mediated by phosphorylation occurring over the few minutes of conditioning (25). In contrast to mechanisms of synaptic homeostatic plasticity, which typically extend over hours and involve gene expression (71, 72), these experiments show changes after only a few minutes, in line with the faster timescales of intrinsic plasticity and our own experiments (73, 74).

K^+^-mediated depolarization techniques are limited in that they afford only population averages. Therefore, this method cannot investigate cell-specific responses to mild depolarization. Also, K^+^-mediated depolarization protocols are not directly translatable to *in vivo* experiments. Nonetheless, we employed these protocols here as they are relatively simple and have reliably identified several signaling and transcriptional events that function in the intact brain in response to sensory stimuli (11, 17, 31, 75, 76). We surmise that the findings of this study may have implications in several aspects of neuronal function and dysfunction. For example, increases in extracellular K^+^ has been hypothesized to be etiologically relevant in epilepsy, migrainous scintillating scotoma, and other forms of cortical spreading depression (77, 78, 79). On the other hand, in normal physiology, if neurons are able to dynamically adjust their intrinsic excitability in response to their activity experience, previous mild depolarization could have an important impact in the memory engram allocation processes. Because a neuron is connected to many other neurons, synchronous firing and subsequent Hebbian potentiation between two neurons in an engram is likely restricted by the intrinsic excitability of connected downstream neurons. Neurons with high intrinsic excitability are more likely to be included in an engram; in corollary, neurons that are left out likely have lower intrinsic excitability (12, 14, 21, 22). Excitability and inclusion are in part determined by competition, wherein excitable neurons actively suppress surrounding competitors *via* intervening inhibitory neurons (14, 15). Enhancing CREB abundance and transcription (including CREB-dependent IEG transcription) also enhances neuronal excitability and engram allocation (12, 18, 21, 80). It is possible, based on our studies, that the recent past subthreshold activity of a given neuron also impacts competition by generating lower intrinsic excitability. In other words, neurons that have experienced mild depolarization may transiently depress responses to subsequent stimulation, thereby predisposing them to exclusion from new engrams. While this possibility remains to be tested in the brain, the idea of recent experiences dictating competitiveness in neuronal networks is nonetheless intriguing.

## Experimental procedures

### Tissue culture

Primary neuronal culture in these experiments was performed as previously described (UC Merced Institutional Animal Care and Use Committee approval: AUP#16-0004) (55). Samples were cortical neurons from E18 embryos plated on 35 cm^2^ dishes or 25 mm^2^ round coverslips (no. 0, Deckglaser #92100100075) and cultured in Neurobasal feeding media with B27–Neurobasal medium (Gibco; catalog no.: 21103049), 25 μM glutamate (Sigma–Aldrich; catalog no.: 1446600), and 0.125x B27 supplement (Gibco; catalog no.: A35828-01). Cells were maintained at 37 °C in a humidified incubator with 5% CO_2_. Half the feeding media was replaced every 3 to 4 days. Neurons were used for assays between 10 and 14 days *in vitro*.

### Cell treatment: two-step paradigm

Isosmotic KCl stock solutions for the primary round of (1°) treatment were prepared at salt concentrations mimicking Neurobasal media where increasing amount of KCl was compensated by decreasing NaCl. Stock solutions (pH 7.4) contained variable KCl (5, 15, 35, and 55 mM; Fisher; P217), 1.8 mM CaCl (Fisher; C79), 0.8 mM MgCl (Fisher; BP214), 26 mM NaHCO_3_ (Fisher; S233), variable NaCl (Fisher; BP358), 1 mM NaH_2_PO_4_ (Fisher; BP330), 25 mM d-glucose (Sigma; G5767), and 11 mM Hepes (Sigma; H3375).

Many experiments conducted for this article were modified versions of our basic two-step protocol. In this protocol, a variable term 1° KCl treatment was followed by a variable term washout stage and then a 15′ secondary round of (2°) treatment with 5 μM Bic. To prepare the 1° KCl treatment, 500 μl of conditioned media was removed from the dish and premixed with 500 μl of the appropriate KCl stock solution in ACSF to achieve the final KCl concentration for application to the cells. KCl solutions were prepared before starting any treatments, and dishes were returned to the incubators while the remaining preparations were completed for treatment. When treatments were ready to begin, no more than four dishes were removed from the incubators at a time. Dishes were treated sequentially, and the researcher noted the exact time of treatment for each dish to keep the timing accurate. When treatment began, old media were removed from the dish with a micropipette and saved. Warm KCl treatment was added to the dish as soon as possible after old media were removed. Dishes were returned to the incubators at the time of KCl treatment. Dishes were removed from the incubator just prior to completion of the 1° stage, and KCl was removed on schedule using a micropipette. For this washout stage, 1 ml of old media from the original dish replaced the KCl solution, and 1 ml of warm conditioned media was added to further dilute any residual KCl. Conditioned media consisted of media drawn on the same day from cultured primary cortical cells the same age and source as the treated cells. Dishes were returned to the incubator for the duration of the washout stage. Dishes were removed from the incubator 2 to 3 min before the end of the washout stage, and media were measured and reduced to 1 ml per dish to prepare for 2° treatment. On schedule, 1 μl DMSO or 5 μM Bic was added to the 1 ml media in the dish for the 2° treatment stage. Each dish was swirled to mix the solution after the addition of 2° treatment, and the collection of treated dishes was returned to the incubator for the duration of the 2° treatment time. Dishes were removed from the incubator just prior to the end of the 2° treatment time, media were removed *via* suction on completion, and appropriate sampling was performed.

### Treatments

#### Bic and 4AP

Neurons were treated with 5 μM Bic (Sigma–Aldrich; catalog no.: 14340) to inhibit GABAergic activity in two-step treatments. The health of the cell culture was assessed in a test sample by treatment with 50 μM Bic treatment and 75 μM 4AP (Acros Organics; catalog no.: 104571000).

#### PMA and TTX

MAPK pathways were activated intracellularly *via* PKC with 1 μM to 1 nM PMA (Sigma–Aldrich; catalog no.: P1585). PMA treatments were applied as a 2° treatment in place of Bic. PMA was diluted in DMSO (Sigma; catalog no.: D2650) to achieve the variable PMA concentrations, so that the same DMSO load was added to each treated condition. PMA treatments were combined with 1 μM TTX (Calbiochem; catalog no.: 554412) to block membrane activity.

#### CHX and FP

Transcription and translation inhibitors were applied from the beginning of treatments at time = 0 and were maintained at the same concentration through all stages of the experiment. Experimental designs contained both inhibitor-added and inhibitor-free conditions for each biological replicate. FP (Sigma; catalog no.: F3055), CHC (Sigma; catalog no.: C7698), and FK506 (Tocris; catalog no.: 3631) treatments (1 μM) were applied from time = 0 at the primary stage for “FK primary” condition, and from time = 1 h 30 min at the start of the secondary stage for the “FK secondary” condition. A control condition was run alongside these treatments, and DMSO was added at each stage of treatment to keep total DMSO additions equivalent across all three conditions. Therefore, all three conditions contained 1 μl DMSO per ml from time = 0 through 1 h 30 min at the start of the 2° stage, and 3 μl DMSO per ml from there on, as the FK secondary condition received FK in 1 μl DMSO and all conditions received 1 μl 5 μM Bic or DMSO as the 2° treatment.

### Electrophoresis and Western blotting

Samples were lifted from cell culture dishes with 75 μl 1× radioimmunoprecipitation assay, made in-house (25 mM Tris–HCl [pH 8]; 150 mM NaCl; 1% sodium deoxycholate; 0.1% SDS; and 0.1% IGEPAL) supplemented with 1:100 protease/phosphatase inhibitor cocktail (Thermo; catalog no.: 78442). Lysates were sheared by sonication (3 × 30 s, lowest setting on Bioruptor). Cell debris was pelleted at 15,000 rpm for 5 min at 4 °C, and clarified supernatant was removed to a new 1.5 ml tube. For Western preparation, equal volumes of the supernatant were used for each sample. Samples were combined with dye (4× Laemmli sample buffer; Bio-Rad; catalog no.: 1610747) with 10% β-mercaptoethanol (Sigma; catalog no.: 63689) and were boiled for 5 min at 95° in a heat block. Sample and dye mixtures were then loaded in a 4 to 20% (Bio-Rad; catalog no.: 4568095) or 4 to 15% (Bio-Rad; catalog no.: 456-1083) Mini PROTEAN gel in Tris–glycine/SDS (Bio-Rad; catalog no.: 1610772). Gels were run at 150 V for ∼10 min and then 110 V until the dye band reached a few centimeters above the end of the gel. Resolved proteins were transferred to a polyvinylidene difluoride membrane (Bio-Rad; catalog no.: 10026933) using the Bio-Rad Trans-Blot Turbo Transfer System with 20% EtOH-containing transblot turbo transfer buffer (Bio-Rad; catalog no.: 10026938) on the mixed molecular weight setting (7 min). Polyvinylidene difluoride membranes were immediately transferred to cold Tris-buffered saline with Tween-20 (TBS-T) and incubated at 4 °C overnight in 1° antibody in 5 ml 1× TBS-T with 1.5% bovine serum albumin (Fisher; catalog no.: BP9703). The pERK antibody (rabbit, Cell Signaling; catalog no.: 4370S) was diluted at 1:1000 dilution, and the beta-actin antibody (mouse; Thermo Fisher Scientific; AM4302) was diluted at 1:10,000 dilution. Membranes were washed three times in 1× TBS-T before being probed with 2° antibody for 45 min at room temperature. 2° antibodies were either goat-anti–mouse 647 (Research Resource Identifier [RRID]: AB 2535808) or goat-anti–rabbit 546 (RRID: AB 2534093) Alexa Fluor secondary antibodies (Life Technologies). Membranes were washed three times with 1× TBS-T for 5 min each and imaged using Bio-Rad Multiplex ChemiDoc Imaging System.

### RNA extraction and gene transcription quantitation with real-time PCR

Total RNA was collected using the illustra RNAspin Mini kit (GE Lifesciences; catalog no.: 25050072). Samples were collected with 350 μl RNA lysis buffer from the kit and after processing were precipitated in 40 μl RNase-free water. Specific pre-mRNAs from total RNA samples were initially amplified by complementary DNA synthesis (14 cycles) using primers overlapping an intron–exon junction and a OneStep RT–PCR kit (Qiagen; catalog no.: 210212). Each reaction used 250 ng of RNA per reaction. The housekeeping transcript was Gapdh. The complementary DNA product was diluted 1:20 with RNase-free water, and 4 μl were used for each qRT–PCR using PerfeCTa SyBR Green FastMix (QuantaBio; catalog no.: 95072-012) and the Bio-Rad CFX Connect real-time PCR Detection System. Samples were run in technical duplicates for each primer, and the average Ct value was used with the ΔΔCt method to calculate fold change.

### [Ca^2+^]_i_ imaging

[Ca^2+^]_i_ was recorded as previously described with minor modifications (81). Cells were loaded by incubation with 5 μM fura-2 AM in 0.04% pluronic acid in cell culture media for 45 to 60 min at 37 °C followed by washing with conditioned media in the absence of indicator for 10 min. For experiments in which the cells were treated with ɷ-contoxins, the toxins were present during fura-2-AM loading but were absent during the wash. Coverslips containing fura-2-loaded cells were transferred to a recording chamber, placed on the stage of an Olympus IX71 microscope and viewed through a 40× objective. Excitation wavelength was selected with a galvanometer-driven monochromator (8-nm slit width) coupled to a 75-W xenon arc lamp (Optoscan; Cairn Research). [Ca^2+^]_i_ was monitored using sequential excitation of fura-2 at 340 and 380 nm; image pairs were collected every 1 s. For experimental recordings, cells were superfused at a rate of 1 to 2 ml/min with Neurobasal salt solution for 2 min followed by 60 s perfusion with Neurobasal salt solution containing 10, 20, or 30 mM KCl as indicated. Fluorescence images (510/40 nm) were projected onto a cooled charge-coupled device camera (Cascade 512B; Roper Scientific) controlled by MetaFluor software (Molecular Devices). After background subtraction, the 340- and 380-nm image pairs were converted to [Ca^2+^]_i_ using the formula [Ca^2+^]_i_ = *K*_*d*_β(*R* − *R*_min_)/(*R*_max_ − *R*) (82). The dissociation constant (*K*_*d*_) for fura-2 was 145 nM. β is the ratio of fluorescence intensity acquired with 380 nm excitation measured in Ca^2+^-free buffer (1 mM EGTA) and buffer containing saturating Ca^2+^ (5 mM). R is 340 nm/380 nm fluorescence intensity ratio. *R*_min_, *R*_max_, and β were determined in a series of calibration experiments on intact cells. *R*_min_ and *R*_max_ values were generated by applying 10 μM ionomycin in Ca^2+^-free buffer (1 mM EGTA) and saturating Ca^2+^ (5 mM), respectively. Values for *R*_min_, *R*_max_, and β were 0.37, 9.38, and 6.46, respectively. These calibration constants were applied to all experimental recordings. The neuronal cell body was selected as the region of interest, and data from all somata in the field were averaged. All neurons within the imaging field were included in the analysis, and no exclusions were made. Each cover glass was imaged once (n = 1).

### Hierarchical clustering of rIEG expression

Pre-mRNA levels of rIEGs under various experimental conditions were used for hierarchical clustering analysis to summarize results and reveal trends in regulation of gene expression. Each gene was represented as a vector of fold-change values (for each time point and treatment group) and organized into matrices for clustering analysis. Specific normalization details are provided in figure legends. Matrices were uploaded to Morpheus (https://software.broadinstitue.org/morpheus), a tool made available through the Broad Institute, which was used to generate expression heatmaps, perform clustering analysis, and export dendrograms. Briefly, the clustering analysis computes the Euclidian distance between genes based on provided features (expression levels under various conditions and time points), which are then used to recursively pair genes into clusters by average linkage, from closest to farthest, generating dendrograms to visualize relatedness.

### MEA experiments

Neurons from the preparations described in the cell culture methods section were plated on poly-l-lysine/laminin-coated MEAs (60MEA200/30-Ti; multichannel systems) in 600 μl of B27-supplemented Neurobasal plating media. Cells were fed every 3 to 4 days by exchanging approximately half the media with B27-supplemented BrainPhys feeding media (StemCell). This was done to promote optimal conditions for neuronal firing, which has been shown to be enhanced in BrainPhys media (83). Recordings were made with an MEA2100-lite system that interfaces with multichannel system provided Multi Channel Experimenter software. Sampling was conducted at 10 kHz in 2-min sessions at room temperature (arrays were covered to prevent contamination).

### MEA data analysis

Recordings were initially postprocessed in multichannel analyzer with a high-pass first-order Butterworth filter with 100 Hz cutoff prior to generation of spike time stamps. Spikes were detected using an automatic threshold estimator set to 5 to 8 standard deviations from the baseline signal depending on the amount of baseline noise. To quantify burst properties, the multichannel analyzer burst detection tool was used with the following settings: maximum interval to start burst, 25 ms; maximum interval to end burst, 250 ms; minimum interval between bursts, 500 ms; minimum duration of burst, 50 ms; and minimum number of spikes in burst, 5. Raster images from example recordings were generated in NeuroExplorer (Nex Technologies). For data presented in Figure 6, each recording was summarized as an average of all electrodes for various parameters (number of spikes, etc.). For data in Figures 7 and 8, electrodes were individually analyzed and displayed, pooling all electrodes for each treatment group across replicates to generate the distributions presented. Individual electrode data were generated from multichannel analyzer and then further processed in R (R Core Team, 2014). Plots and statistics were generated using GraphPad Prism, version 8.4.2 (GraphPad Software, Inc).

### Statistics of qRT-PCR and Western blot

Data were analyzed using GraphPad Prism 7 (RRID: SCR_002798). Where possible, normality was assessed with the Shapiro–Wilk test and reported, though ANOVA was carried out regardless as it is somewhat robust to deviations from normality. Outliers were identified for each cell of the design using ROUT, Q = 5%. All analyses were run with and without outliers. Results for all outliers included are reported, and any differences when outliers were excluded are noted. Data were analyzed with appropriate three-way, two-way, or one-way ANOVAs. Three- and two-way interactions were considered first. When insignificant, we next considered two-way interactions and main effects, respectively. When interactions were significant, we reorganized the data to investigate simple main effects by collapsing over insignificant factors and/or by separating the data by each level of one factor for analysis. Prism does not automatically use the pooled error term from the larger ANOVA comparison, so for main effects and simple main effects analyses following up on larger ANOVAs, we ran the reorganized data using the next ANOVA down. For example, for three-way ANOVAs with insignificant three-way interactions but a significant two-way interaction, we reorganized the data by collapsing over the insignificant factor and ran an explicit two-way ANOVA. We then followed the same procedure for the two-way ANOVA, running a one-way ANOVA or unpaired *t* test for simple main effects at each level of a factor if the two-way interaction was significant, or, for main effects, by collapsing the data again over the insignificant factor and running a one-way ANOVA or unpaired *t* test on the entire dataset. Multiple comparisons were reported for the lowest-level ANOVA, and the Bonferroni correction (CHECK) was applied to adjust the significance. Mean differences are reported with the 95% confidence interval and significance value. Alpha levels were set to 0.05. Error bars represent standard error of the mean throughout, except where otherwise noted. Biological replicates are indicated throughout as N in corresponding figure legends. Biological replicates constitute cell culture preparations from the pooled cortices of embryos from independent litters.

## Data availability

All data are summarized and presented in the article. Furthermore, the corresponding author will entertain requests for any dataset(s).

## Conflict of interest

The authors declare that they have no conflicts of interest with the contents of this article.
